# A comprehensive in silico analysis of non-synonymous and regulatory SNPs of human *MBL2* gene

**DOI:** 10.1186/s40064-016-2543-4

**Published:** 2016-06-21

**Authors:** Namarta Kalia, Aarti Sharma, Manpreet Kaur, Sukhdev Singh Kamboj, Jatinder Singh

**Affiliations:** Department of Molecular Biology and Biochemistry, Guru Nanak Dev University, Amritsar, 143005 India; Department of Human Genetics, Guru Nanak Dev University, Amritsar, India

**Keywords:** Candidate gene studies, Computational analysis, Mannose binding lectin gene 2, Non-coding SNPs, Non-synonymous SNPs, Single nucleotide polymorphism (SNP)

## Abstract

Mannose binding lectin (MBL) is a liver derived protein which plays an important role in innate immunity. Mannose binding lectin gene 2 (*MBL2*) polymorphisms are reported to be associated with various diseases. In spite of being exhaustively studied molecule, no attempt has been made till date to comprehensively and systematically analyze the SNPs of *MBL2* gene. The present study was carried out to identify and prioritize the SNPs of *MBL2* gene for further genotyping and functional studies. To predict the possible impact of SNPs on MBL structure and function SNP data obtained from dbSNP database were analyzed using various bioinformatics tools. Out of total 661 SNPs, only 37 validated SNPs having minor allele frequency ≥0.10 were considered for the present study. These 37 SNPs includes one in 3′ near gene, nine in 3′ UTR, one non-synonymous SNP (nsSNP), thirteen intronic SNPs and thirteen in 5′ near gene. From these 37 SNPs, 11 non-coding SNPs were identified to be of functional significance and evolutionary conserved. Out of these, 4 SNPs from 3′ UTR were found to play role in miRNA binding, 7 SNPs from 5′ near and intronic region were predicted to involve in transcription factor binding and expression of *MBL2* gene. One nsSNP Gly54Asp (rs1800450) was found to be deleterious and damaging by both SIFT and Polyphen-2 servers and thus affecting *MBL2* protein stability and expression. Protein structural analysis with this amino acid variant was performed by using I-TASSER, RAMPAGE, Swiss-PdbViewer, Chimera and I-mutant. Information regarding solvent accessibility, molecular dynamics and energy minimization calculations showed that this variant causes clashes with neighboring amino acids residues that must interfere in the normal triple helix formation of trimeric subunit and further with the normal assembly of MBL oligomeric form, hence decrease in stability. Thus, findings of the present study indicated 12 SNPs of *MBL2* gene to be functionally important. Exploration of these variants may provide novel remedial markers for various diseases.

## Background

Mannose binding lectin (MBL) is a liver derived acute phase protein. It binds to carbohydrates on the surface of mannose-rich pathogens and mediates clearing by phagocytosis or complement activation (Nepomuceno et al. 1997; Selander et al. 2006). Mice have two homologous genes of MBL i.e. *MBL1* and *MBL2*, but in human the first of them is pseudogene leaving one functional gene *MBL2* (Guo et al. 1998). The *MBL2* gene is composed of 7461 bases and lies between the regions 52765380 to 52772841 bp of chromosome no. 10 (NCBI reference sequence number NC_000010.11). The gene contains four exons and three introns. It encodes a 248 amino acid residue transmembrane protein, MBL (NCBI accession number XP_011538118.1) which is encoded via a 3570 bp long mRNA (NCBI accession number NM_000242.21). It belongs to a family of proteins called collectins, which consists of collagenous region and a carbohydrate recognition domain (Taylor et al. 1989). MBL consists of multimers of an identical polypeptide chain of 32 kDa.

A single-nucleotide polymorphism (SNP) is the most common type of genetic variation. Several studies have shown *MBL2* SNPs of promoter and exonic region regulate the *MBL2* serum levels in different autoimmune diseases and infectious diseases, including HIV infection, leishmaniasis, leprosy, malaria, schistosomiasis, tuberculosis and trypanosomiasis (Madsen et al. 1995; Summerfield et al. 1995; Garred et al. 1997; Kelly et al. 2000; Klabunde et al. 2000; Jack and Turner 2003; IP et al. 2005; Garred et al. 2006; Alonso et al. 2007). SNPs of *MBL2* gene cover both coding and non-coding regions. However, not all the coding region elements are functionally important. Only the non-synonymous SNPs (nsSNPs), also called as missense variants are particularly important as they result to changes in the translated amino acid residue sequence. nsSNPs may affect the protein function by reducing protein solubility or by destabilizing protein structure (Chasman and Adams 2001). Moreover, analyses on conserved non-coding region have shown that non-coding DNA is involved in biological functions (Alexander et al. 2010). These non-coding elements can have various regulatory functions within the genome, such as interacting with transcription factors (TFs), miRNA, creating splice sites and acting as exonic splicing enhancers (ESEs) (Birney et al. 2007). Despite their important regulatory role, much less effort has been invested in the functional analysis of non-coding SNPs for candidate gene studies as compared to the coding regions SNPs.

There are several publically available databases for SNPs, such as dbSNP, GWAS Central, SwissVar etc. dbSNP is the most extensive among all the databases (Sherry et al. 2001; Bhagwat 2010). It contained a total of 661 SNPs in the *MBL2* gene of *Homo sapiens* as of October, 2015. To date the functional significance has not been established for the majority of them. In the absence of any experimental information on their functional effects, the potential functional consequences of a SNP can be predicted using various bioinformatics tools (Bhatti et al. 2006; Johnson 2009; Li and Wei 2015). These tools predict the functional effects of SNPs at five main levels: splicing, transcriptional, translational, post-translational and protein stability. The majority of current bioinformatics tools examine the functional effects of SNPs only with respect to a single biological function. However, the others provide a comprehensive assessment of SNP function based on different algorithms, data and resources (Bhatti et al. 2006; Johnson 2009; Li and Wei 2015).

All the SNPs present in the *MBL2* gene were analysed using various composite and singleton tools to verify their putative functional effects. The SNPs that were identified as having functional effects were then prioritized on the basis of number of criteria; i.e. the significance of the function identified, presence within an evolutionary conserved region, validation status of the SNP, and the minor allele frequency of the SNP. Thus, the present study involves filtering through a list of SNPs to identify causal variants. The study was further explored to view the effect of nsSNP on the stability of MBL protein. To the best of our knowledge, this is the first comprehensive computational study undertaken for in silico analysis of nsSNPs as well as regulatory SNPs in *MBL2* gene.

## Methods

**Fig. 1:**
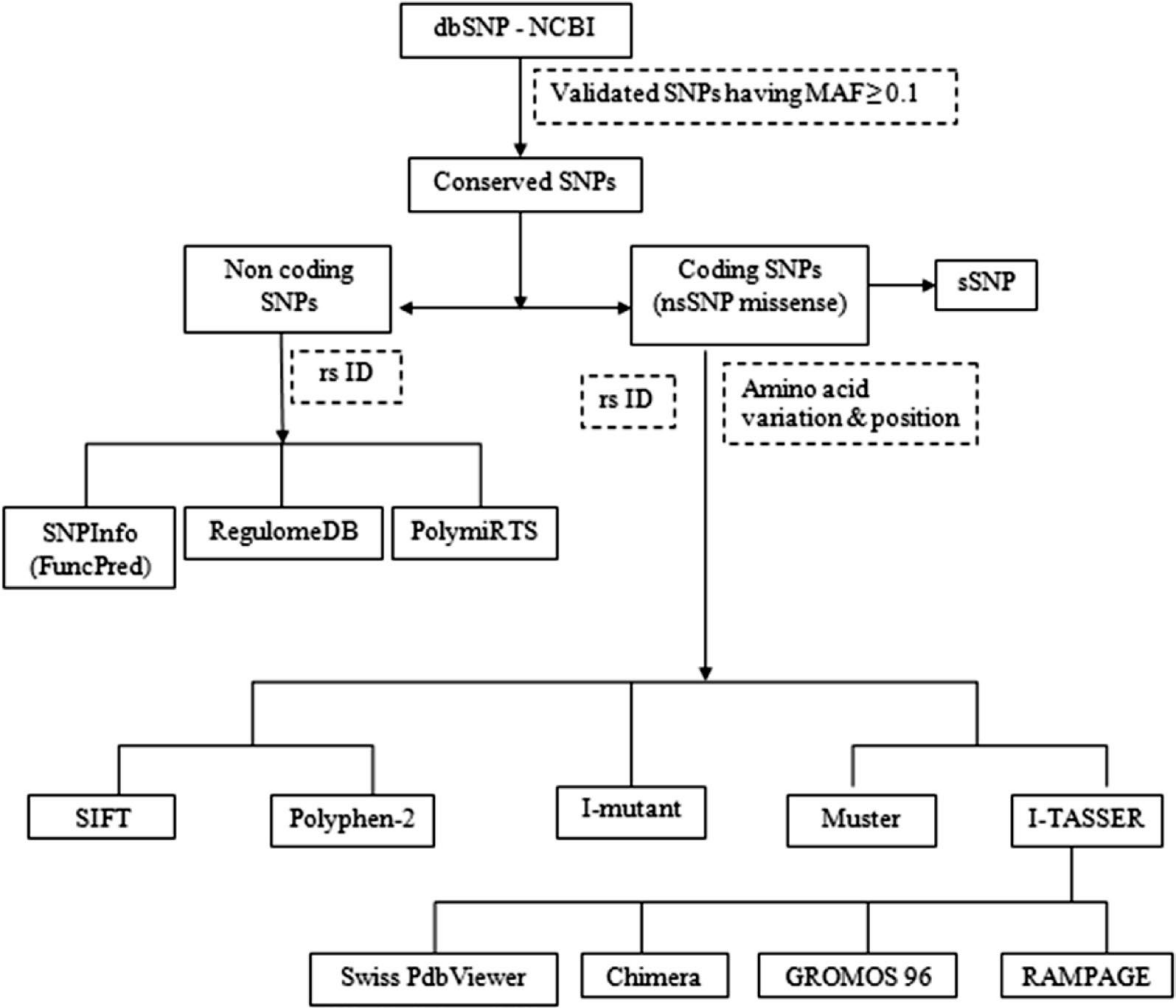
Schematic representation of computational tools for in silico analysis of *MBL2* gene

The SNPs and their related protein sequence for the *MBL2* gene were obtained from dbSNP (http://www.ncbi.nlm.nih.gov/SNP/) and were subjected to various computational analyses. The strategy followed to select SNPs having structural and functional impact is shown in Fig. 1.

### Identifying SNPs located in evolutionary conserved regions in the gene

SNPs located in evolutionary conserved regions (cSNPs) were identified using the Ense*mbl* Genome browser release 48 (http://www.ensembl.org/). *MBL2* gene from seventeen eutherian mammals was selected for the comparative analysis with the human *MBL2* gene. These includes *Homo sapiens* (Human), *Pan troglodytes* (Chimpanzee), *Gorilla gorilla gorilla* (Gorilla), *Pongo abelii* (Orangutan), *Chlorocebus sabaeus* (Vervet-AGM), *Macaca mulatta* (Macaque), *Papio Anubis* (Olivebaboon), *Bos Taurus* (Cow), *Ovis aries* (Sheep), *Sus scrofa* (Pig), *Equus caballus* (Horse) *Felis catus* (Cat), *Canis lupus familiaris* (Dog), *Callithrix jacchus* (Marmoset), *Mus musculus* (Mouse), *Oryctolagus cuniculus* (Rabbit) and *Rattus norvegicus* (Rat). For a detailed comparison, base pair view was selected from the database and the SNPs were verified manually.

### Non-coding SNP functional analysis

To identify the effect of SNPs in non-coding regions, tools predicting potential functional effect of SNPs at Transcription factor binding sites (TFBS), Intron/exon border consensus sequences (splice sites), Exonic splicing enhancers (ESEs), and miRNA binding were used.

*SNPinfo (FuncPred) and RegulomeDB* provide a collection of functional information using variety of tools and resources. The SNPs functionality was primarily determined by SNPinfo (FuncPred) (http://snpinfo.niehs.nih.gov/snpinfo/snpfunc.htm) web server in Asian population (Xu and Taylor 2009). This webserver helps in selecting SNPs for genetic association studies and consists of 3 pipelines for SNP selection and a composite tool for SNP function prediction named ‘FuncPred’. Which is composite of Polyphen, SNP3D, MATCH, TRANSFAC 12.1, rescue ESE, ESEfinder, FAS–ESS, miRanda and miRBase. A user can query results for all the SNPs in a gene or chromosomal region, or for a list of input SNPs. For the present study, a list of SNP rsIDs was uploaded for batch analysis with default settings. The output was a list of SNPs with possible functional effect.

To complement SNP prioritization, SNPs were further analysed by RegulomeDB (http://regulomedb.org/). It is an online composite database and prediction tool to annotate as well as prioritize potential regulatory variants from human genome (Boyle et al. 2012). The database includes up-to date high quality datasets from Encyclopedia of DNA Elements transcription factor, chromatin immunoprecipitation sequencing (ChIP-seq), histone ChIP-seq, Formaldehyde-Assisted Isolation of Regulatory Elements, DNase I hypersensitive site data and other sources like large collection of Expression quantitative trait loci, dsQTL, ChIP-exo data to identify putative regulatory variants. RegulomeDB classifies variants into six categories ranging from 1 to 6, where category 1 variants are ‘likely to affect binding and linked to expression of a gene target’, category 2 variants are ‘likely to affect binding’, Category 3 variants are ‘less likely to affect binding’, and Category 4, 5 and 6 variants have ‘minimal binding evidence’. RegulomeDB also assigns a score of 7 for variants with no annotation data available. dbSNP rsIDs were used as input for the present study. A list of rsIDs allotted with different categories and also with no data available was obtained.

*PolymiRTS (v 3.0)* is an integrated database that systematically identifies DNA polymorphisms in miRNAs and miRNA target sites (PolymiRTS). It also elucidates the potential links of SNPs to molecular, physiological, behavioral and disease phenotypes (Bhattacharya et al. 2014). This computational server was used in order to determine the effect of 3′ UTR SNPs of *MBL2* gene in creating and abolishing miRNA target sites resulting in diverse functional consequences. Gene symbol was selected, *MBL2* was entered as search information and submitted to PolymiRTS (v3.0) (http://compbio.uthsc.edu/miRSNP/). A list of all 3′ UTR SNPs of *MBL2* gene, creating or destructing miRNA site was obtained. These 3′ UTR SNPs were further looked for possible miRNA IDs for which they create and abolish site for.

### Non-synonymous SNP functional analysis

Deleterious and damaging effect of nsSNP was predicted using web based tools SIFT (Sorting Intolerant from Tolerant) and Polyphen-2 (Polymorphism Phenotyping v2).

*SIFT* (http://sift.bii.a-star.edu.sg/) performs analysis based on different algorithms and it interprets the homologous sequences using the Swiss-Prot (version 51.3) and TrEMBL (version 34.3) (Kumar et al. 2009). Results were expressed as SIFT scores which were classified as damaging (0.00–0.05), potentially damaging (0.051–0.10), borderline (0.101–0.20), or tolerant (0.201–1.00). nsSNP rsID were uploaded to get the possible results.

*Polyphen***-***2* (http://genetics.bwh.harvard.edu/pph2/) was used to predict the possible impact of an amino acid substitution on both structure and function of protein by analysis of multiple sequence alignment and protein 3D structure (Adzhubei et al. 2013). Protein sequence, database ID/accession number, amino acid position and amino acid variant details are the input options for PolyPhen-2. Protein sequence with NCBI accession number XP_011538118.1 was input for the present study. Prediction outcomes could be classified as probably damaging, possibly damaging or benign according to the score ranging from (0–1). “Score” is the probability of the substitution being damaging; “sensitivity” and “specificity” correspond to prediction confidence. The predicted damaging effect is also indicated by a vertical black marker inside a color gradient bar, where green is benign and red is damaging.

### Protein structure prediction

Protein blast (http://blast.ncbi.nlm.nih.gov/Blast.cgi?PAGE=Proteins) was used to find the proteins related to the *MBL2*. To obtain a list of the closest matches it performs a FASTA search against every macromolecular structure deposited in protein data bank (PDB) (http://www.rcsb.org/pdb/home/).

*Muster v1.0* (http://zhanglab.ccmb.med.umich.edu/MUSTER/) provides the Z-score and complete full length models by using Modeller v8.2 (Wu and Zhang 2008). The corresponding template is considered good if the calculated Z-score is greater than 7.5.

*I***-***Tasser* (http://zhanglab.ccmb.med.umich.edu/I-TASSER/) server provides accurate structural and functional predictions using state-of-art algorithms (Roy et al. 2010). It reports up to five models which correspond to the five largest structure clusters. The confidence of each model is quantitatively measured by C-score (confidence score), calculated based on the significance of threading template alignments and the convergence parameters of the structure assembly simulations. C-score typically lies in the range of −5 to 2 where a higher value signifies a model with a higher confidence and vice versa. TM-score (template modeling score) >0.5 highlights a model of correct topology and a TM-score <0.17 indicates a random similarity. It also predict solvent accessibility with values range from 0 (buried residue) to 9 (highly exposed residue). Protein sequence with NCBI accession number XP_011538118.1 was input for the present study. Structural evaluation was carried out using *RAMPAGE* ramachandran plot analysis (http://mordred.bioc.cam.ac.uk/~rapper/rampage.php) (Lovell et al. 2003).

### Modeling nsSNP on protein structure

*Swiss***-***PdbViewer (v4.10)* was used to generate the mutated models of the selected protein struture for the corresponding amino acid substitutions (Guex and Peitsch 1997). It replaces the native amino acid with the variant. The.pdb files were saved for the model. This server also uses Gromacs as the default force field for energy minimization calculations.

*Chimera* (http://www.cgl.ucsf.edu/chimera) is a extensible molecular modeling system used for interactive visualization and analysis of molecular structures and related data, including density maps, supramolecular assemblies, sequence alignments, docking results, trajectories and conformational analysis (Pettersen et al. 2004).

### Protein stability prediction

*I***-***Mutant 2.0* (http://folding.uib.es/i-mutant/i-mutant2.0.html), a neural network based tool, predicts the change in the stability of the protein upon mutation (Capriotti et al. 2005). This tool automatically predicts protein stability changes upon single site mutations. Prediction can be performed using either protein structure or sequence. The FASTA sequence of protein with NCBI accession number XP_011538118.1 retrieved from NCBI is used as an input to predict the mutational effect on protein stability. Output obtained is in the form of protein stability change upon mutation and Gibbs-free energy change (DDG).

## Results

**Fig. 2:**
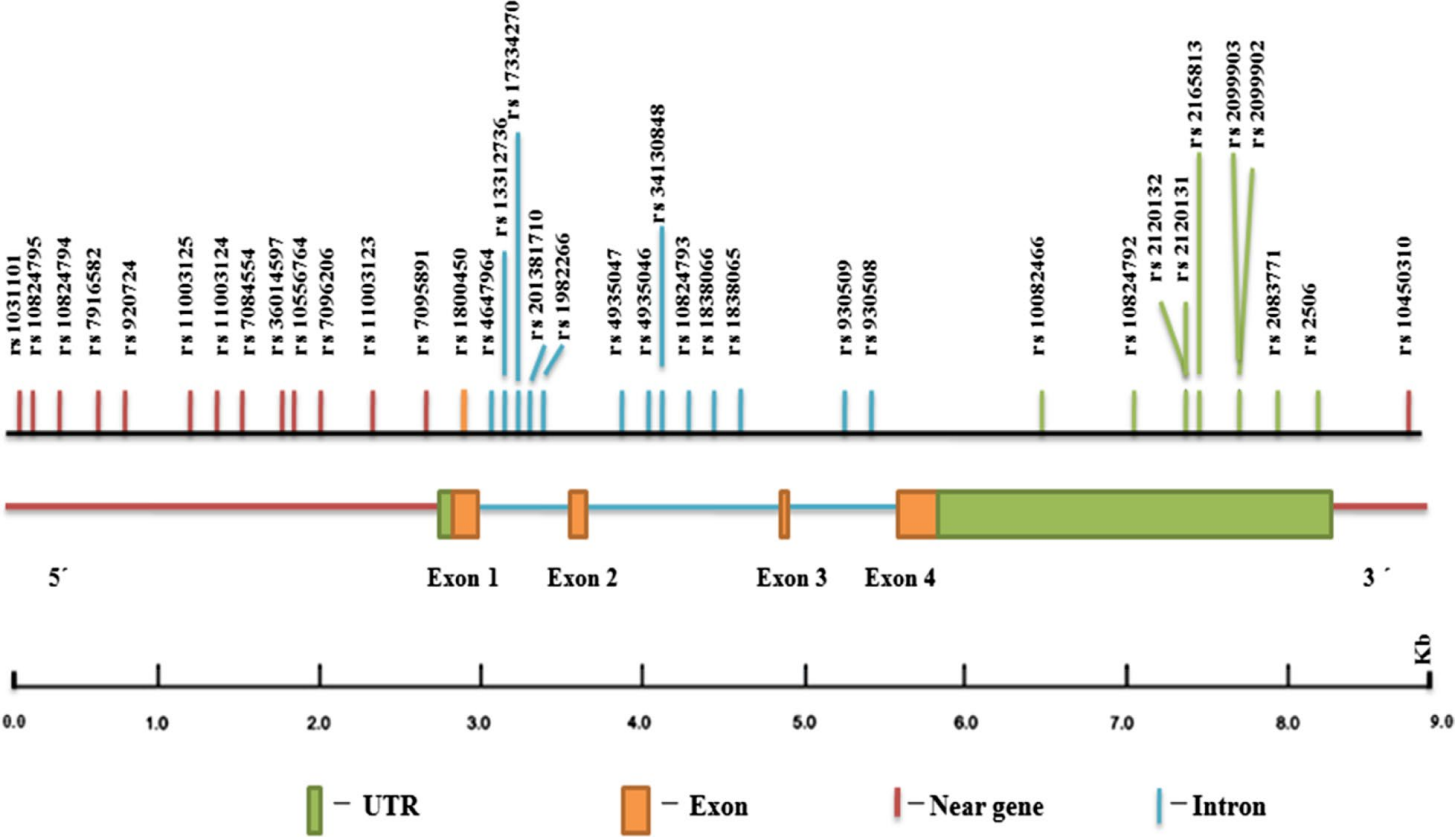
A map of the *MBL2* gene highlighting the positions of SNPs (MAF ≥ 0.10) based on dbSNP database. Genomic structure of entire *MBL2* gene contains 4 exons and spans 9.0 kb. Approximate distances of various regions of *MBL2* gene are indicated in bp underneath

The dbSNP database contains both validated and non-validated polymorphisms. In spite of this drawback, dbSNP was availed because it is the most extensive SNP database (Sherry et al. 2001; Bhagwat 2010). *MBL2* gene contains 661 SNPs in dbSNP database. Out of these 661 SNPs, only 37 validated SNPs having MAF ≥ 0.10 were considered for the present study. Those SNPs which either have MAF < 0.10 or are not validated were excluded for further analysis. These 37 SNPs includes one 3′ near gene, nine 3′ UTR, thirteen intronic SNPs, thirteen 5′ near gene and one synonymous SNP (sSNP). One sSNP was excluded from the study leaving 36. However no non-synonymous SNPs were found to have MAF even ≥0.05. So, SNPs falling in the list of pathogenic significance were also checked for validation status and MAF ≥ 0.1. Only one SNP Gly54Asp (rs1800450) have MAF = 0.1220 was found and hence included in the study. Thus, our investigation accounted for 37 SNPs including 3′ UTR, intronic, 5′/3′ near gene and nsSNP. A map of the *MBL2* gene region highlighting the positions of each of these SNPs is shown in Fig. 2.

A list of 37 SNPs was submitted to the FuncPred program and results obtained are shown in Table 1. Total 24 SNPs were predicted to have effect on function, of which nine 3′ UTR SNPs were found to affect miRNA binding site and three intronic and twelve 5′ SNPs were found to be affecting transcription binding site. RegulomeDB database has divided 37 SNPs included in the present study into six broad categories (Category 1 to Category 6)—where twenty nine SNPs had annotation scores in between 1 and 6 (Table 2) and the remaining eight SNPs had no data annotation (not shown in table). Of the 29 SNPs, only one SNP had RegulomeDB score of 1f three SNPs were less likely to be functional (Category 3a) while, 25 SNPs had minimum functional evidence (Category 4, 5 and 6). The top ranked SNP rs7096206 had annotation for eQTL + TF binding/DNase peak and thus very likely to have regulatory functions.

**Table 1:** List of non-coding SNPs predicted by FuncPred as having functional effect

Region	SNP ID	Chr. position	MAF	TFBS	miRNA
3′ UTR	rs2506	52765749	0.3389	–	✓
3′ UTR	rs2083771	52765918	0.3391	–	✓
3′ UTR	rs2099902	52766089	0.3387	–	✓
3′ UTR	rs2099903	52766097	0.2732	–	✓
3′ UTR	rs2165813	52766224	0.2742	–	✓
3′ UTR	rs2120131	52766258	0.2742	–	✓
3′ UTR	rs2120132	52766280	0.2734	–	✓
3′ UTR	rs10824792	52766446	0.4519	–	✓
3′ UTR	rs10082466	52766862	0.2770	–	✓
Intron	rs1982266	52770876	0.4774	✓	–
Intron	rs17334270	52771083	0.2835	✓	–
Intron	rs13312736	52771221	0.2835	✓	–
5′ near gene	rs7095891	52771701	0.2833	✓	–
5′ near gene	rs11003123	52771774	0.2837	✓	–
5′ near gene	rs7096206	52771925	0.1955	✓	–
5′ near gene	rs36014597	52772040	0.2837	✓	–
5′ near gene	rs7084554	52772053	0.2831	✓	–
5′ near gene	rs11003124	52772131	0.2835	✓	–
5′ near gene	rs11003125	52772254	0.3061	✓	–
5′ near gene	rs920724	52773037	0.2953	✓	–
5′ near gene	rs7916582	52773235	0.2841	✓	–
5′ near gene	rs10824794	52773429	0.2841	✓	–
5′ near gene	rs10824795	52773533	0.2841	✓	–
5′ near gene	rs1031101	52773600	0.1222	✓	–

*MAF* minor allele frequency, *TFBS* transcription factor binding site, ✓ SNPs which affect function, – SNPs which does not affect function

**Table 2:** List of SNPs predicted by regulomeDB score

dbSNP ID	RDB score	Category	Description
rs7096206	1f	Likely to affect binding and linked to expression of a gene target	eQTL + TF binding/DNase peak
rs930508	3a	Less likely to affect binding	TF binding + any motif + DNase peak
rs10556764			
rs7084554			
rs930507	4	Minimal binding evidence	TF binding + DNase peak
rs930509			
rs13312736			
rs7095891			
rs11003123			
rs36014597			
rs10450310	5	Minimal binding evidence	TF binding or DNase peak
rs10824792			
rs10082466			
rs35137523			
rs1838065			
rs1838066			
rs34130848			
rs4935046			
rs17287498			
rs17334270			
rs4647964			
rs11003124			
rs11003125			
rs34130848			
rs2099902	6	Minimal binding evidence	Motif hit
rs2120131			
rs1982266			
rs10824794			
rs1031101			

*RDB* RegulomeDB, *TF* transcription factor, *eQTL* expression quantitative trait loci

**Fig. 3:**
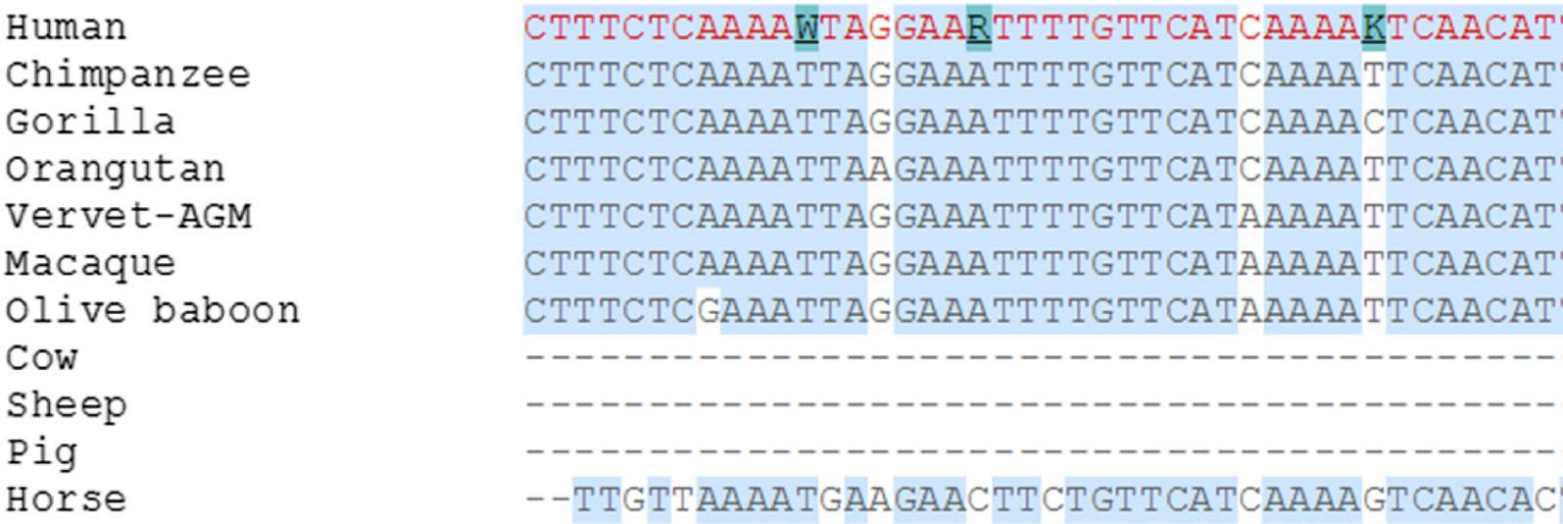
A comparison of the human *MBL2* gene with eutherian mammals: the screenshot compares human chromosome no. 10: 52764780-52770775 bp region with homologous regions of other mammals. Human *MBL2* gene sequence indicated in *red color*. Conserved region are represented by *blue* highlighted region. The highlighted nucleotide indicates SNPs. The screen shot was taken from Ense*mbl* Genome browser release 48. Only species showing alignment with human *MBL2* gene are shown in figure

In sequence alignment, except 6 species including *F.catus*, *C. lupus familiaris*, *C. jacchus*, *M. musculus*, *O. cuniculus* and *R. norvegicus*, all were found to show alignment with the human *MBL2* gene. Furthermore, all the 37 SNPs involved in present study were found to lie in conserved region, hence are called conserved SNPs (cSNPs) (Fig. 3). 3′ UTR SNPs submitted online to PolymiRTS server showed four variants affecting miRNA target sites, hence proposed to have obvious consequences on protein truncation (Table 3). These four SNPs were also predicted by FuncPred for miRNA binding. The 3′ UTR SNPs identified as having putative functional effects, by the four tools were then screened as shown in Table 4. Two SNPs predicted by all the tools and two SNPs i.e. rs2099903, rs2165813, not predicted by regulomeDB but by other 3 tools, were selected for further analysis because prediction by polymiRTS cannot be ignored.

**Table 3:** List of 3′ UTR SNPs analyzed by PolymiRTS

dbSNP ID	Alleles	miRNA motif
rs2099902	G	hsa-miR-797
rs2099903	G	hsa-miR-4666a-5p/hsa-miR-8060
	T	hsa-miR-1252-3p/hsa-miR-3646
rs2165813	C	hsa-miR-1253/hsa-miR-6770-5
	T	hsa-miR-452-5p/hsa-miR-4676-3p/hsa-miR-4738-3p/hsa-miR-539-5p/hsa-miR-548 g-3p/hsa-miR-892c-3p
rs10082466	T	hsa-miR-4719/hsa-miR-513b-5p
	C	hsa-miR-27a-3p/hsa-miR-27b-3p/hsa-miR-3185/hsa-miR-513a-5p

**Table 4:** List of 3′ UTR SNPs screened based on all the tools used

SNP ID	FuncPred	RegulomeDB score	cSNPs	PolymiRTS	Prioritize
rs2099902	✓	6	✓	✓	*
rs10082466	✓	5	✓	✓	*
rs2099903	✓	–	✓	✓	*
rs2165813	✓	–	✓	✓	*
rs2120131	✓	6	✓	–	–
rs10824792	✓	6	✓	–	–
rs2506	✓	–	✓	–	–
rs2083771	✓	–	✓	–	–
rs2120132	✓	–	✓	–	–

*cSNPs* conserved SNPs, ✓ SNPs which affect function, – SNPs which does not affect function, * prioritized for further studies

Similarly, intronic and 5′ near gene SNPs identified as having putative functional effects, by the three tools were screened as shown in Table 5. From the SNPs identified by all the three tools only 7 SNPs which were reported and have regulomeDB score ≤5 were selected and those which either were not reported (rs17334270, 13312736) or have regulomeDB score ≥6 (rs10824794, rs1031101, rs1982266) were not selected. Thus overall total 11 non-coding SNPs form 3′ UTR and 5′ near region of *MBL2* gene were filtered through a plethora of SNPs and identified as causal variants. Predictions of deleterious effect of rs1800450 (nsSNP) was performed by SIFT and Polyphen-2 softwares. It was predicted to be damaging by both the servers. The SIFT prediction was deleterious with a score of 0.006. This deleterious nsSNP was submitted to Polyphen-2 as query protein sequences in FASTA format with prediction outcomes to be probably damaging score closer to 1.

**Table 5:** List of non-coding SNPs except of 3′ UTR screened based on all the tools used

Region	Snp ID	FuncPred	RegulomeDB score	cSNPs	Selected
5′ near gene	rs7095891	✓	4	✓	*
	rs11003123	✓	4	✓	*
	rs7096206	✓	1f	✓	*
	rs10556764	–	3a	✓	–
	rs36014597	✓	4	✓	*
	rs7084554	✓	3a	✓	*
	rs11003124	✓	5	✓	*
	rs11003125	✓	5	✓	*
	rs920724	✓	–	✓	–
	rs7916582	✓	–	✓	–
	rs10824794	✓	6	✓	–
	rs10824795	✓	–	✓	–
	rs1031101	✓	6	✓	–
Intronic	rs1982266	✓	6	✓	–
	rs17334270	✓	5	✓	–
	rs13312736	✓	4	✓	–
	rs4647964	–	5	✓	–
	rs1838065	–	5	✓	–
	rs1838066	–	5	✓	–
	rs34130848	–	5	✓	–
	rs4935046	–	5	✓	–
	rs930508	–	3a	✓	–
	rs930509	–	4	✓	–

cSNPs conserved SNPs, ✓ SNPs which affect function, – SNPs which does not affect function, * prioritized for further studies

**Fig. 4:**
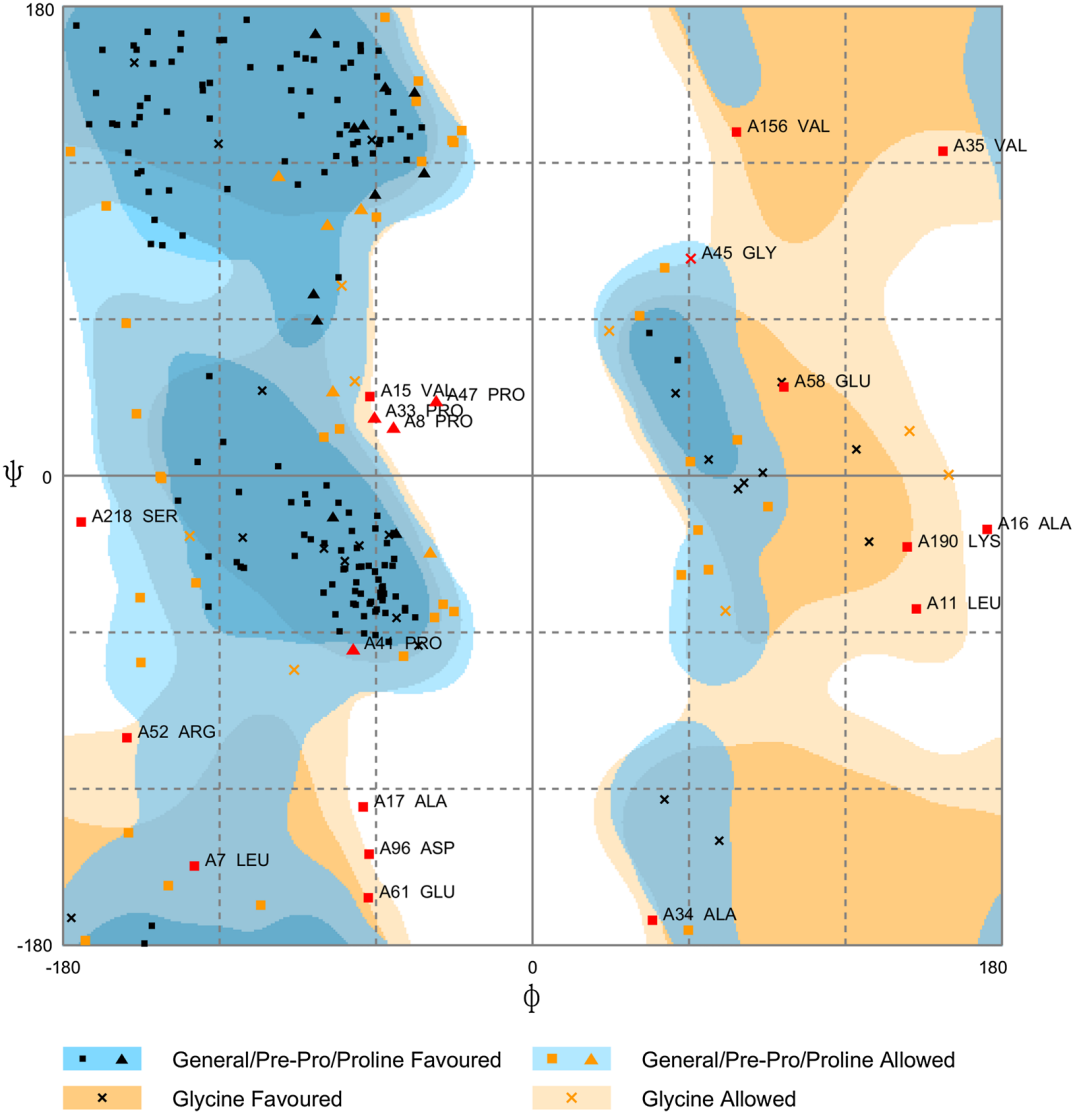
Ramachandran plot of predicted MBL secondary structure

By using Research Collaboratory for Structural Bioinformatics Protein Data Bank (RCSB PDB), the *MBL2* gene related protein structures were searched. Although different mutations in *MBL2* gene are reported, the native primary structure (with complete 248 amino acid sequence) of MBL monomeric protein is not yet available. Only partial protein structure, with PDB ID 1HUP was found (Sheriff et al. 1994). This partial structure of *MBL2* is a 148-residue peptide, stretches from 107 to 248 aa residues consisting of the ‘neck’ and carbohydrate recognition domains forming trimers in solution and in crystals. MUSTER provided highest score for the same template (PDB ID 1HUP) with Z score 13.966 and alignment length 141 aa residues with coverage of 0.568. However, the rs1800450 nsSNP lies in collagenous domain of human MBL protein (Super et al. 1989; Summerfield et al. 1997; Wallis and Cheng 1999; Wallis 2002; Turner 2003; Larsen et al. 2004). Hence, protein modelling based on its structural information was necessary for absolute understanding of its functionality. I-TASSER was chosen to predict MBL monomeric protein secondary structure. Five models were obtained as an output, of these first model with highest C score of −2.80, TM score of 0.39 ± 0.13 and estimated RMSD 12.4 ± 4.3 Å was considered for the analysis of highly damaging nsSNPs on the structure and function of protein. The Ramachandran plot was also assessed to support quality of predicted MBL model using RAMPAGE server (Fig. 4). The model showed good proportions of residues in favored (72.4 %), allowed (19.9 %) and outlier regions (7.7 %). The results indicated that the 3D model was of fair quality. I-TASSER also predicted that native Gly residue with score of 3 as buried residue and variant Asp residue with score of 7 as highly exposed in terms of solvent accessibility.

**Fig. 5:**
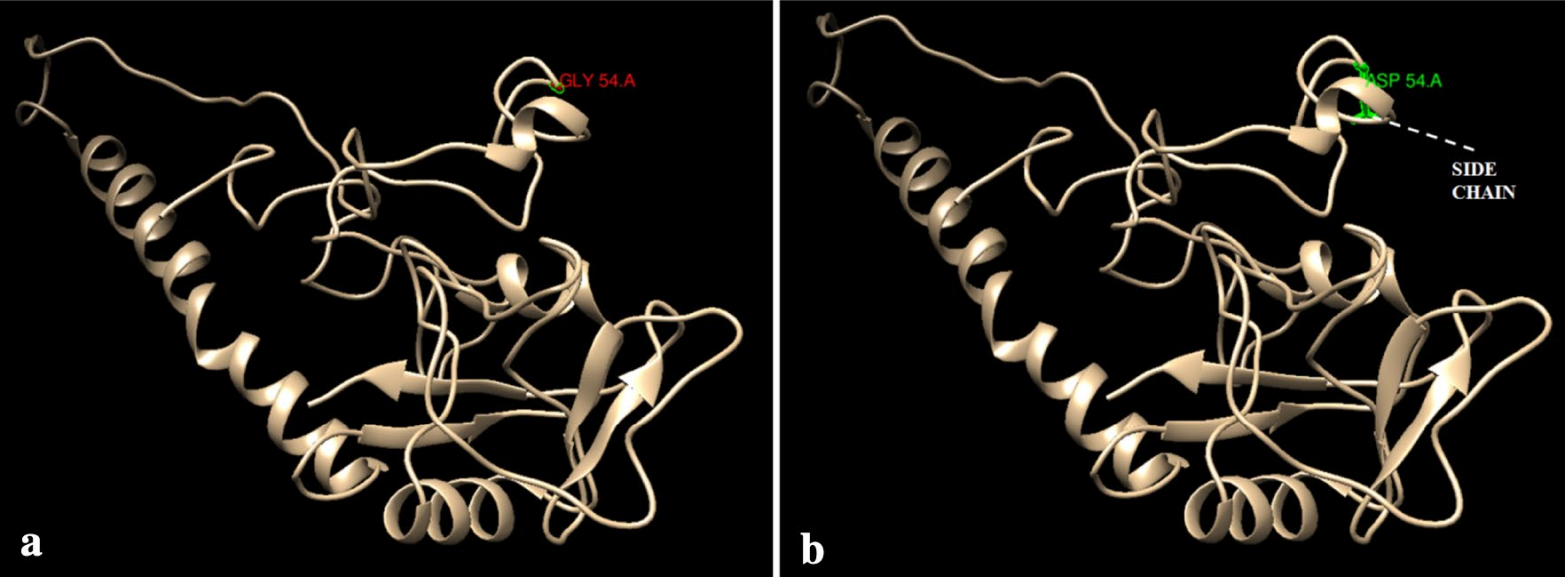
Full length models of MBL protein monomer with **a** native residue glycine (*red colored* and *labeled*) at position 54, **b** variant residue aspartic acid (*green colored* and *labeled*) with side chain indicated by *dotted white lines*

**Fig. 6:**
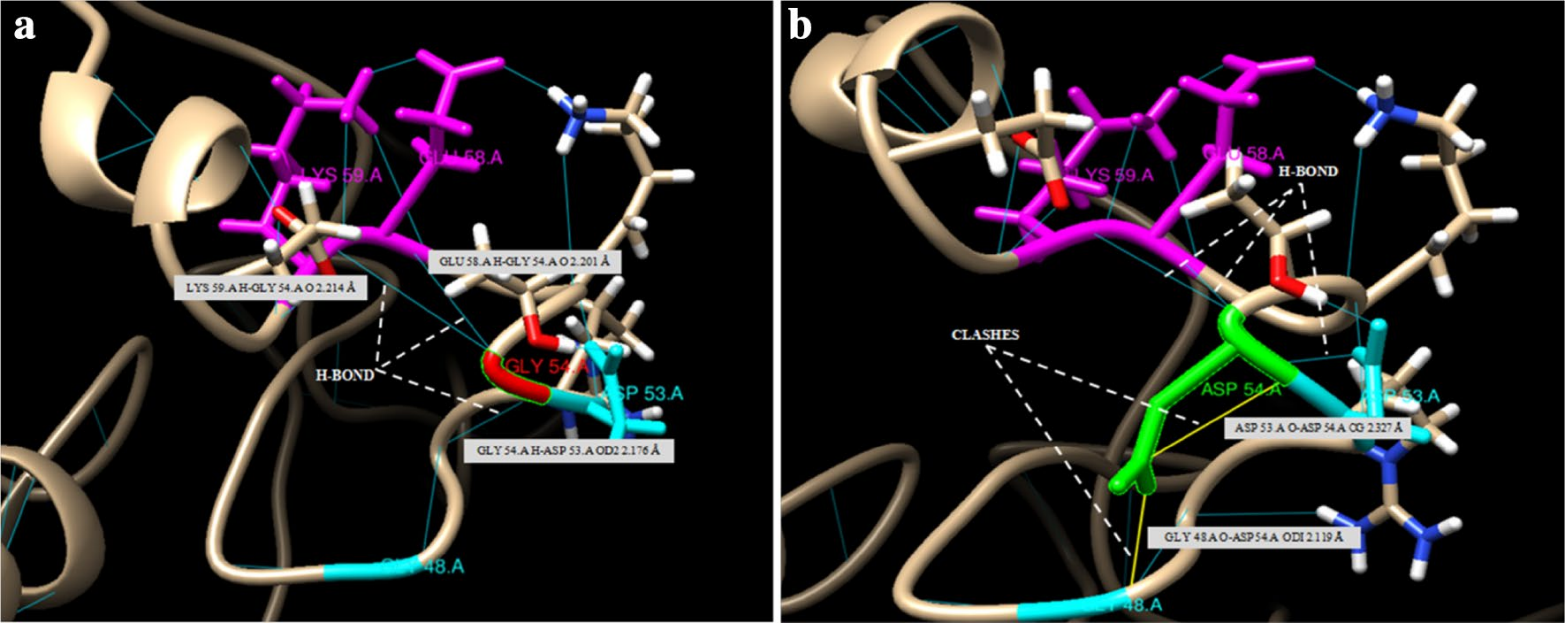
3D analysis of wild and variant residues at position 54 **a** Gly (*red color*) at 54 position, forms three H-bonds (*blue lines*, indicated with *white dotted lines*) with nearby residues Asp 53 (*sky-blue color*), Glu 58 and Lys 59 (*purple color*). **b** Network of clashes (*yellow lines*, indicated with *white dotted lines*) appeared between variant residue Asp 54 (*green color*) with Gly 48 and Asp 53 (*sky-blue color*)

To get variant modeled structure at position 54 i.e. Gly → Asp conversion Swiss-PdbViewer and chimera was used (Fig. 5). This variation leads to slight deviation in various parameters including total energy after minimization, total electrostatic constraint, total bond energy, torsion and non-bonded energy (Table 6). It was found that Gly → Asp conversion at position 54 showed a network of clashes with nearby residues that is Asp at position 53 and Gly at position 48 as shown in Fig. 6 while this network is lacking when native residue glycine was there. Furthermore, analysis by I-mutant indicated that Gly → Asp conversion at position 54 decreases the stability of protein with DDG value of −1.37 kcal/mol.

**Table 6:** Parameters of native and mutant structure after energy minimization

Parameters	Native amino acid structure	Variant amino acid structure
Total energy (KJ/mol)	−5854.403	−5852.157
Total electrostatic constraint	−6399.19	−4496.11
Total bond energy (KJ/mol)	1833.712	1834.508
Torsion (KJ/mol)	2475.780	2485.522
Non-bonded energy (KJ/mol)	−6399.19	−6385.21

## Discussion

Mannose binding lectin (MBL) is a pattern recognition molecule of the innate immune system. It plays an important role in the first line of defense and provides primary immune response to pathogens and thus is important when adaptive immune response will not operate due to some reasons (Ip et al. 2009). Studies have indicated association of some SNPs of *MBL2* gene with susceptibility to various diseases as well as were shown to affect serum MBL levels (Madsen et al. 1995; Summerfield et al. 1995; Garred et al. 1997; Kelly et al. 2000; Klabunde et al. 2000; Jack and Turner 2003; IP et al. 2005; Garred et al. 2006; Alonso et al. 2007). However, there are large numbers of SNPs present in the human *MBL2* gene that have been identified. But it is difficult to study all the SNPs present in a gene for candidate gene approach as some SNPs may have no functional effect or of very low frequency. As the interpretation of clinically important novel variants often remain challenging, many bioinformatics tools have been developed that predict biological consequences of these polymorphisms. The present study has hauled out some novel SNPs of MBL protein along with prediction of biological effect of some already studied SNPs using these tools. Thus focus can be put on studying those SNPs which probably may have important role in various diseases. Variants of non-coding and coding regions are equally important and hence are the focus of the present study (Alexander et al. 2010; Birney et al. 2007).

To identify most likely functional variants of *MBL2* gene, of which many are still unknown, in silico analyses was used. However, no single bioinformatics tool can be used to obtain a complete picture of the functional significance of allelic variants. Hence the current analysis was conducted using a number of complementary bioinformatic tools. As expected the results obtained from different tools were not directly overlapping for all the studied SNPs. Four composite tools SNPinfo, Genomic alignments by Ensembl, regulomeDB and poylmiRTS were used for the analysis of non-coding SNPs. The entire four tools played equal important role in prioritizing functional SNPs leading to selection of 11 non-coding SNPs for disease association studies. Two of the most widely accepted tools SIFT and PolyPhen-2 was used to predict the phenotypic effect of nsSNP on the physico-chemical properties of the concerned proteins and thus expression. Both the tools predicted rs1800450, a deleterious and damaging single nucleotide variant.

Out of 12 SNPs, 4 SNPs with rs numbers rs11003125, rs7095891, rs7096206 and rs1800450 are very well known and a plethora of literature is available regarding their functional and deleterious effect and their association with various diseases including filariasis, malaria, leishmaniasis, leprosy, tuberculosis, trypanosomiasis, HIV infection, systemic lupus erythematosus, rheumatoid arthritis (Choi et al. 2001; Boldt et al. 2006; Garred et al. 2006; Asgharzadeh et al. 2007; de Messias-Reason et al. 2007; Ip et al. 2009; Meyrowitsch et al. 2010; Panda et al. 2013; Martiny et al. 2012; Singla et al. 2012; Weitzel et al. 2012; Li et al. 2013; Chen et al. 2014; Jha et al. 2014). rs1800450 (Gly 54 Asp) variation leads to the production of nonfunctional monomers that further interfere with the formation of higher MBP oligomers, leading to alterations in the functional activity of the protein and their circulating levels (Sumiya et al. 1991; Lipscombe et al. 1992; Madsen et al. 1994; Terai et al. 2003; Larsen et al. 2004). Three SNPs in Promoter region rs7095891, rs7096206 and rs11003125 are functionally characterized to alter the transcriptional levels significantly contributing the large variation in the promoter activity of the *MBL2* gene elucidating their biological significance with respect to gene expression and hence the serum MBL levels (Naito et al. 1999b; Juliger et al. 2000). The combined effect of these polymorphisms on MBL basal expression and serum levels was also studied (Madsen et al. 1995; Garred et al. 2006).

Furthermore, a single study has indicated that rs2099902, rs10082466 variants of 3′-UTR and functional secretor haplotypes in *MBL2* are associated with increased colon cancer risk in African Americans. The C allele of rs10082466 exhibited a binding affinity of miR-27a and this allele was associated with both lower MBL plasma levels and activity (Zanetti et al. 2012). So validation of these SNPs by these reports complements the finding of the present study. However, very scanty data is available regarding role of rs11003123, rs36014597, rs7084554, rs11003124 SNPs of *MBL2* gene only a single study had shown the association of rs11003124 with leprosy in Han Chinese population (Boldt et al. 2009; Swale et al. 2014; Wong et al. 2012; Zhang et al. 2013). Furthermore, no clinical relevance of rs2165813 and rs2099903 SNP is documented till date. Thus, more epidemiological and clinical studies are needed to validate the SNPs reported in the present study.

MBL is a complex of six sets of homotrimers of a monomer containing 228 amino acids (Ezekowitz et al. 1988; Sastry et al. 1989; Taylor et al. 1989; Kurata et al. 1994). This monomer consists of four domains a 20-amino acid N-terminal cysteine-rich domain, a collagen-like domain consisting of 18–20 tandem repeats of Gly-Xaa-Yaa, an alpha helical coiled-coil neck region, and a carbohydrate recognition domain. The neck region initiates the folding and the collagen-like region zips toward the N terminus, creating trimeric subunits. Interchain disulphide bonds link the N terminal domains of polypeptides together both within and between subunits to form and stabilize higher oligomers (Wallis and Drickamer 1999). Human MBL comprises dimers to hexamers of trimeric subunits of which trimers and tetramers are probably the predominant from in circulation (Lu et al. 1990; Teillet et al. 2005). However, a variant monomer may result into the formation of nonfunctional, low-order oligomers with shortened half life (Lipscombe et al. 1995; Naito et al. 1999a; Petersen et al. 2001).

Although *MBL2* gene is widely known because of its important role and association with many diseases, still the full native protein structure is not yet available in protein data bank (PDB). Native protein structure is important for the in silico analysis of functional polymorphisms. As already explained in results, only partial structure, with PDB ID 1HUP corresponding to neck and carbohydrate recognition domains of human MBL is available (Sheriff et al. 1994). Gly54Asp variation lies in the collagenous domain of human MBL, therefore, I-TASSER was used for predicting 3D secondary structure of native MBL monomeric protein that includes all the four domains along with the signal peptide and analyzed for damaging mutation predicted by SIFT and polyphen-2. The TM-score of the present study predicted structure (>0.5) which represents correct topology for protein while higher value of C-score signifies model with a higher confidence represents good quality protein. Ramachandran plot also supported the quality of monomeric MBL protein model.

The comparison of specific properties of native and variant structures revealed difference in stability. This variation leads to slight deviation in energy and decrease in stability. This change in stability can be explained by the reason that the variant residue has bulkier R group than the wild type and cannot fit within the available space. The free carboxylic acid group on Asp can disturb the ionic interaction of nearby residues and hence causing vulnerable effects. The wild-type residue is non-polar while the variant residue is negatively charged and hydrophilic. Furthermore, there is difference in solvent accessibility of both residues with native being buried and variant being fully exposed, hence slight deviation in energy and decrease in stability.

This variation which lies in the fifth Gly-Xaa-Yaa repeat distort the collagen-like region had been shown to interfere with normal assembly of the MBL oligomeric form and it’s interactions with MBL associated serine proteases (Wallis and Cheng 1999; Wallis and Dodd 2000; Turner 2003; Larsen et al. 2004; Wallis et al. 2004). Some studies have suggested that the MBL triple helix is formed when the variant state has an enthalpy similar to the native state but with reduced stability (Wallis and Cheng 1999; Wallis and Dodd 2000; Wallis et al. 2004). The structural and energetic consequences of Gly → Asp conversion were also investigated by synthesizing two triple-helical peptides containing Gly/Asp at position 54 (Mohs et al. 2005). The study indicated loss of triple helix content by Gly → Asp replacement accompanying with small decrease in stability of triple helix. However, no enthalpy change was observed. Furthermore NMR studies indicated the complete distortion of triple helix in peptide containing variant residue. Hence, providing evidence in support of predictions made by SIFT, Polyphen-2, I-mutant and clashes that was observed during Gly → Asp conversion in the theoretical modeling of MBL monomeric peptide.

In the present study, we have used the relatively simple structure i.e. monomeric polypeptide of MBL to examine and understand the effect of this variation on its more complex homolog using theoretical modeling. Extensive studies had been carried out to investigate the effect of Gly substitution in the collagen like domain using synthetic peptides which offer an approach to characterizing the effect of mutations on the triple-helix (Baum and Brodsky 1999). Bella with other colleagues reported the high-resolution structure of a (Pro-Hyp-Gly)_10_ peptide with a Gly to Ala substitution near the center indicated a local loss of direct hydrogen bonding and a slight untwisting at the mutation site when the three collagen like peptide chains form a triple helix in solution (Bella et al. 1994). Similar effort was made in the present study using in silico tools where we used the theoretical 3D model of MBL monomer polypeptide to visualize the effect of Gly → Asp variation at position 54 and the sequence surrounding the replacement and it was found that Asp at position 54 showed a network of clashes with nearby residues while this network was lacking when the native residue glycine was there. Thus the clashes observed must interfere in the normal triple helix formation of trimeric subunit and further with the normal assembly of MBL oligomeric form.

There are some essential features that are used in this study to avoid the common pitfalls while conducting an in silico analysis. Feature one, looking for validation status to check the reliability of a given SNP as all SNPs in dbSNP are not real. Some polymorphisms might have arisen solely due to sequencing errors and others may be sole to the individuals (Fredman et al. 2002). Second important feature is prioritizing these SNPs that lie within an evolutionary conserved region to remove false-positive predictions that encountered due to in silico tools (Fairbrother et al. 2002; Cartegni et al. 2003; Yeo and Burge 2004). The high rate of false positive findings produced by in silico prediction tools can be due to the short length of sequences (typically 6–8-mer) used in computer simulations (Yeo and Burge 2004). Thus, this strategy was used in the current study to validate the in silico predictions.

Third and very important feature to prioritize functional SNPs identified with in silico tools is the use of MAF. Because MAF is linked to statistical power of the study i.e. MAF and sample size to detect variant allele of a SNP in a given sample population have inverse relationship (Grover et al. 2007). Therefore, SNPs with a MAF of 0.05 or more are generally targeted in the majority of large scale genome studies for instance the international HapMap project. Keeping this in view, MAF of 0.10 or more were used for the present analysis. Thus by keeping in mind these features and based on the results of tools 12 SNPs out of 661 SNPs of *MBL2* were found to be functionally important for candidate gene studies.

## Conclusion

The present study demonstrated that 12 SNPs in the *MBL2* gene are functionally important as well as deleterious to its structure and expression. These conserved SNPs may broaden our understanding of genotype phenotype relationship. Out of these 12 SNPs only 4 SNPs i.e. rs11003125, rs7095891, rs7096206 and rs1800450 are widely studied. But the rest of 8 functional SNPs rs2099902, rs10082466, rs11003123, rs36014597, rs7084554, rs11003124, rs2165813, rs2099903 reported by the present study haven’t been explored much till date. Thus, the findings of the present study provide a guideline to the fellow researchers to know important role of these SNPs in the etiology of complex diseases.

## Abbreviations

ChIPseq: chromatin immunoprecipitation sequencing
cSNPs: conserved SNPs
Eqtl: expression quantitative trait loci
ESEs: exonic splicing enhancers
MBL: mannose binding lectin
*MBL2*: mannose binding lectin gene
nsSNP: non-synonymous SNPs
PDB: protein data bank
Polyphen-2: polymorphism phenotyping v2
RAMPAGE: ramachandran plot analysis
SIFT: sorting intolerant from tolerant
SNP: single nucleotide polymorphism
TFBS: transcription factor binding sites
TFs: transcription factors
UTR: untranslated regions
